# Weighted gene coexpression network analysis and machine learning reveal oncogenome associated microbiome plays an important role in tumor immunity and prognosis in pan-cancer

**DOI:** 10.1186/s12967-023-04411-0

**Published:** 2023-08-12

**Authors:** Shi-Wei Guan, Quan Lin, Xi-Dong Wu, Hai-Bo Yu

**Affiliations:** 1 Department of Hepatobiliary Surgery, Wenzhou Central Hospital, The Dingli Clinical Institute of Wenzhou Medical University, Wenzhou, 325000 Zhejiang People’s Republic of China; 2 Department of Neurosurgery Surgery, Wenzhou Central Hospital, The Dingli Clinical Institute of Wenzhou Medical University, Wenzhou, 325000 Zhejiang People’s Republic of China

**Keywords:** Tumor microbiome, Tumor genome, TCGA, WGCNA, Tumor microenvironment, Prognosis, TP53, Tissierella

## Abstract

**Background:**

For many years, the role of the microbiome in tumor progression, particularly the tumor microbiome, was largely overlooked. The connection between the tumor microbiome and the tumor genome still requires further investigation.

**Methods:**

The TCGA microbiome and genome data were obtained from Haziza et al.’s article and UCSC Xena database, respectively. Separate WGCNA networks were constructed for the tumor microbiome and genomic data after filtering the datasets. Correlation analysis between the microbial and mRNA modules was conducted to identify oncogenome associated microbiome module (OAM) modules, with three microbial modules selected for each tumor type. Reactome analysis was used to enrich biological processes. Machine learning techniques were implemented to explore the tumor type-specific enrichment and prognostic value of OAM, as well as the ability of the tumor microbiome to differentiate TP53 mutations.

**Results:**

We constructed a total of 182 tumor microbiome and 570 mRNA WGCNA modules. Our results show that there is a correlation between tumor microbiome and tumor genome. Gene enrichment analysis results suggest that the genes in the mRNA module with the highest correlation with the tumor microbiome group are mainly enriched in infection, transcriptional regulation by TP53 and antigen presentation. The correlation analysis of OAM with CD8+ T cells or TAM1 cells suggests the existence of many microbiota that may be involved in tumor immune suppression or promotion, such as *Williamsia* in breast cancer, *Biostraticola* in stomach cancer, *Megasphaera* in cervical cancer and *Lottiidibacillus* in ovarian cancer. In addition, the results show that the microbiome-genome prognostic model has good predictive value for short-term prognosis. The analysis of tumor TP53 mutations shows that tumor microbiota has a certain ability to distinguish TP53 mutations, with an AUROC value of 0.755. The tumor microbiota with high importance scores are *Corallococcus*, *Bacillus* and *Saezia*. Finally, we identified a potential anti-cancer microbiota, Tissierella, which has been shown to be associated with improved prognosis in tumors including breast cancer, lung adenocarcinoma and gastric cancer.

**Conclusion:**

There is an association between the tumor microbiome and the tumor genome, and the existence of this association is not accidental and could change the landscape of tumor research.

**Supplementary Information:**

The online version contains supplementary material available at 10.1186/s12967-023-04411-0.

## Background

With millions of lives affected and enormous economic and social costs, cancer remains the world’s most burdensome disease [1]. Traditional ideas about tumor research have focused on tumor cells or self-factors such as the tumor microenvironment (TME) and have not considered the role of the microbiome in tumors. Recently, as tumor research has expanded, it has been found that even tumor tissues previously thought to be sterile harbor a microbiome, albeit in low abundance. Most microbes do not appear to cause cancer directly but rather participate in cancer progression [2–4]. Poore et al. showed that the bacterial composition of tumor tissue was about 0.68%, corresponding to about 10^5^ to 10^6^ bacteria per accessible 1 cm^3^ tumor or about 34 bacteria/mm^2^ in a three-dimensional or flat environment [5, 6]. The tumor microbiome plays an important role in cancer progression, diagnosis, treatment, chemoresistance and regulation of immune activity [7–10]. For example, in breast cancer (BRCA), the tumor microbiome can accompany tumor cells across the circulatory system and promote distant colonization of tumor cells [11]. In pancreatic cancer (PAAD), tumor microbes expressing the bacterial enzyme cytidine deaminase degrade gemcitabine leading to chemotherapy resistance [8]. However, the interaction and causal relationship between the tumor microbiome and genome are still unclear.

Tumor Protein P53 (TP53) is a key tumor suppressor gene that is mutated in more than 50% of human cancers [12]. The TP53 signaling pathway responds to various stress signals and regulates a transcriptional program that contributes to tumor suppression. TP53 is involved in many biological processes, such as stem cell formation, metabolism, and regeneration [13–15]. Moreover, mutant TP53 gains oncogenic functions that are independent of wild type TP53, such as promoting invasion, migration, angiogenesis, chemoresistance, and mitotic defects, in addition to losing its original function [16, 17]. However, the association between TP53 mutations and tumor microbes in pan-cancer has not been reported in detail recently.

Research has shown that microbiome is associated with oncogenome. *Fusobacterium nucleatum*’s (*F. nucleatum*) FadA adhesin binds to E-cadherin on colorectal cancer (CRC) cells’ surface, facilitating *F. nucleatum*’s attachment and invasion of epithelial cells. This activates Wnt/β-catenin signalling, which leads to Checkpoint Kinase 2 (CHK2) upregulation, causing Deoxyribonucleic Acid (DNA) damage and promoting CRC progression [18–20]. Furthermore, TP53 mutations are only carcinogenic in the presence of microbially produced gallic acid; otherwise, they have a protective effect in vivo and in organs [21]. In lung cancer, cells with the Kirsten rats arcomaviral oncogene homolog (Kras) mutations and TP53 deletion do not produce lung cancer in germ-free or antibiotic-treated mice [22]. These studies all indicate that microbiome and the tumor genome are related and that this link affects tumor progression.

Weighted gene coexpression network analysis (WGCNA) can identify gene sets that work together and potential biomarkers or therapeutic targets based on the gene set’s endogeneity and its association with the phenotype [23]. WGCNA can also reveal associations between microbiome data and host phenotypes [24, 25]. However, few studies have applied WGCNA to tumor microbiomes. As for machine learning (ML), it is undeniable that ML has had a huge impact on biology. It refers to the process of fitting predictive models to data or identifying groups of information in data [26]. Biological datasets have become larger and more complex in recent years, making ML methods for big data more important and widely used in almost every field of biology. For example, ML can infer the spatial structure of proteins from amino acid sequences, process single-cell data, and predict the prognosis of tumor patients [27–29].

Therefore, we used WGCNA to construct separate networks for the Cancer Genome Atlas (TCGA) tumor microbial data and messenger ribonucleic acid (mRNA) data, and found that the tumor microbiome was extensively correlated with genomic alterations. We defined the microbiome module associated with the oncogenome as oncogenome associated microbiome module (OAM). OAM is tumor-specific enriched. ML methods showed that oncogenome combined with OAM had a better prognostic value. Moreover, tumor microiome could distinguish between TP53 mutations and non-mutations across cancers. Finally, we identified a microorganism, *Tissierella*, commonly found in human feces, belonging to phylum *Firmicutes* and order *Clostridiales*, that may be involved in tumor suppression [30]. Some literature reports have linked *Tissierella* to opportunistic infections in chronic osteomyelitis, arthritis, and liver abscesses [31, 32]. However, studies on the relationship between *Tissierella* and tumors are scarce, and our analysis indicated a potential anticancer effect of *Tissierella*.

## Methods

### Data access and processing

Tumor microbiome counts data for 32 tumor types from TCGA project were obtained from Haziza et al. (https://github.com/knightlab-analyses/mycobiome) [9]. Only data from primary tumors were used. Recent studies have highlighted the important role of intratumor fungi and bacteria in cancer [3, 9, 33]. Therefore, tumor bacteria and fungi were screened for further analysis. To reduce contamination of sequencing plates, which is a common challenge for TCGA tumor microbiome data, we applied the “decontam” R package to perform in silico decontamination of bacterial and fungal data separately [34]. Microbiome data at the genus level were integrated using the “phyloseq” R package [35]. Poore et al. and Haziza et al. reported a significant batch effect for TCGA tumor microbiome data [3, 9]. They proposed a pipeline that transformed discrete taxonomical counts into log-counts per million (log-cpm) per sample using Voom and performed supervised normalization (SNM) [36, 37]. Their study showed that this pipeline increased the biological signal and reduced batch effects of TCGA tumor microbial data. The same pipeline was followed by us to normalize our data. The results of our study suggest that the Voom-SNM pipeline is successful in mitigating the confounding effects resulting from the experimental strategy and data submitting center (Additional file 1: Fig. S1).

TCGA pan-cancer genomic counts data were obtained from the University of Cingifornia Sisha Cruz (UCSC) Xena database (https://xenabrowser.net/datapages/) [38]. Log-cpm transformation was applied to the counts data and 16,370 mRNA that were expressed across all TCGA cancer types were selected for further analysis [39]. TCGA patient survival data and mutation data were also downloaded from “PanCanAtlas Publications” (https://gdc.cancer.gov/about-data/publications/pancanatlas). Cell-type identification by estimating relative subsets of RNA transcripts (Cibersort) immune infiltration data for pan-cancer were acquired from Thorsson et al. [40].

### Construction of tumor microbiome and tumor mRNA WGCNA network

**Fig. 1:**
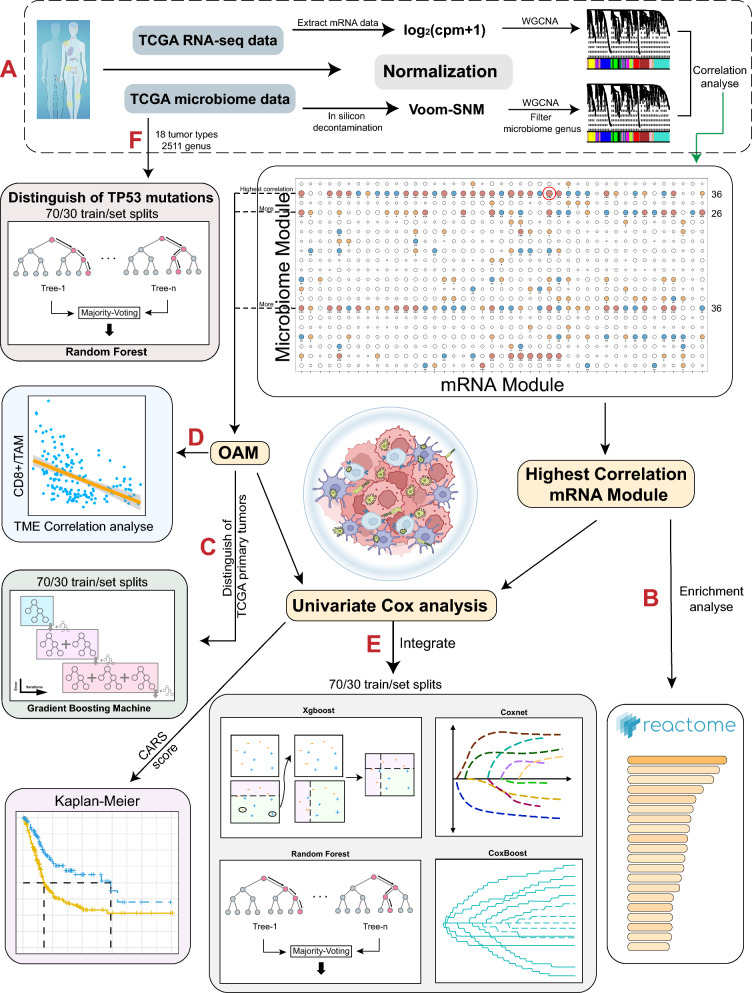
The workflow of our study. **A** After log-cpm and Voom-SNM normalization of TCGA genome and microbiome data, respectively, WGCNA networks were separately constructed and the correlation between their modules was analyzed. **B** The mRNA modules with the highest correlation to microbiome modules in each type of tumor were extracted for Reactome gene enrichment analysis. **C** The OAM of each type of tumor was tested for its ability to discriminate among primary tumors using the gradient boosting machine algorithm. **D** Analysis of microorganisms in OAM for correlation with CD8+ T cells or TAM1 cells. **E** Prognosis-related OAM microbes and prognosis-related mRNAs with the highest correlation to microbiome modules were integrated for each tumor type using four machine learning algorithms: “Coxnet”, “Random Forest”, “Xgboost”, and “Coxboost” for survival analysis. The KM curves demonstrate the prognostic value of the prognostic microorganism with the highest CARS score in each tumor. **F** The relative abundance of 2511 tumor microorganisms at the genus level was included for TP53 wild-type and mutant differentiation at the pan-cancer level. *log-cpm* log-counts per Million, *TCGA* the cancer genome atlas, *WGCNA* weighted gene coexpression network analysis, *mRNA* messenger ribonucleic acid, *OAM* oncogenome associated microbiome module, *CD* cluster of differentiation, *TAM1* tumor-associated macrophages 1, *CARS* correlation-adjusted regression survival, *TP53* tumor protein P53

The uneven data of 32 types of TCGA tumor microbiome needed to be filtered to construct a scale-free network using WGCNA. This was done by calculating the total counts of each microbial genus for each tumor type and applying appropriate thresholds. However, too few microbial genera were left after filtering to be suitable for WGCNA for some tumor types such as PAAD, adrenocortical cancer (ACC), mesothelioma (MESO) and testicular cancer (TGCT). Therefore, we finally included only 18 different types of tumors from BRCA, endometrioid cancer (UCEC), cervical cancer (CESC), lung squamous cell carcinoma (LUSC), lung adenocarcinoma (LUAD), kidney clear cell carcinoma (KIRC), glioblastoma (GBM), head and neck cancer (HNSC), thyroid cancer (THCA), bladder cancer (BLCA), stomach cancer (STAD), colon cancer (COAD), prostate cancer (PRAD), esophageal cancer (ESCA), ovarian cancer (OV), rectal cancer (READ), kidney papillary cell carcinoma (KIRP), and kidney chromophobe (KICH) for follow-up analysis (Fig. 1).

**Fig. 2:**
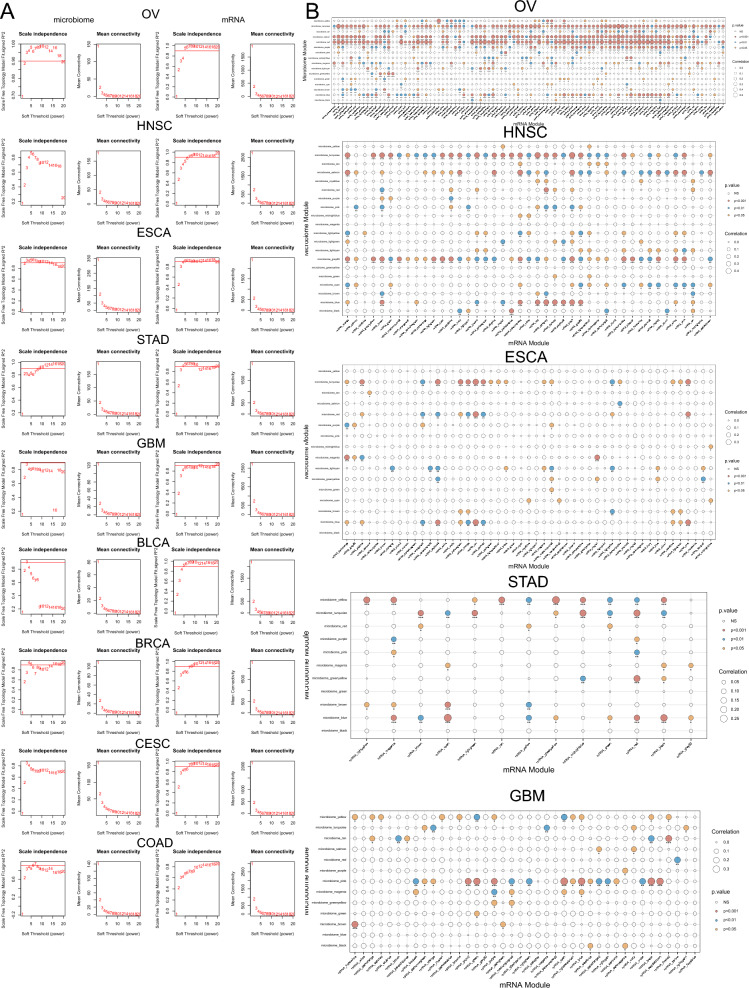
Separate WGCNA networks were constructed for the tumor microbiome data and genomic data. **A** Network topology analysis of various soft threshold powers in OV, HNSC, ESCA, STAD, GBM, BLCA, BRCA, CESC and COAD in microbiome (left two columns) and genome (right two columns). The first column panel shows the scale-free fit index (y-axis) as a function of the soft-thresholding power(x-axis) in the microbiome data. The second column panel displays the mean connectivity (degree, y-axis) as a function of the soft-thresholding power (x-axis) in the microbiome data. The third column panel shows the scale-free fit index (y-axis) as a function of the soft-thresholding power (x-axis) in the genomic data. The forth column panel displays the mean connectivity (degree, y-axis) as a function of the soft-thresholding power (x-axis) in the genomic data. The selection of the soft threshold β is shown in Additional file 5: Table S1. **B** Correlation analysis between the microbial (y-axis) and mRNA (x-axis) modules in OV, HNSC, ESCA, STAD and GBM. The circle size represents the correlation between the microbiome module and the genome module. A p value < 0.05 was considered statistically significant. Red, blue, and yellow circles represent p values less than 0.001, 0.01, and 0.05, respectively. *WGCNA* weighted gene coexpression network analysis, *OV* ovarian cancer, *HNSC* head and neck cancer, *ESCA* esophageal cancer, *STAD* stomach cancer, *GBM* glioblastoma, *BLCA* bladder cancer, *BRCA* breast cancer, *CESC* cervical cancer, *COAD* colon cancer, *mRNA* messenger ribonucleic acid

Normalized microbial genus data and corresponding tumor mRNA data from TCGA were used to construct WGCNA modules for 18 tumor types. (Fig. 2A, Additional file 2: Fig. S2A, Additional file 5: Table S1). A soft threshold β was applied to achieve a scale-free network and the weighted adjacency matrix was converted into a topological overlap matrix. WGCNA soft threshold β selection is the process of choosing a suitable parameter β for constructing a WGCNA that conforms to the features of a scale-free network. A scale-free network is one where the degrees of the nodes follow a power-law distribution, meaning that only a few nodes have many connections, while most nodes have very few. This network structure is biologically meaningful and can capture the coexpression patterns among genes. The WGCNA package provides a function pickSoftThreshold that computes the mean connectivity and the fit index R^2^ of the network for different β values. In general, a β value that makes the mean connectivity between 10 and 100 and the fitted R^2^ value close to 0.8 or higher is reasonable. Therefore, we use the output of the pickSoftThreshold function to determine the appropriate soft threshold β value. Then, modules were identified using the dynamic tree cutting method and the gray module was excluded as recommended by the WGCNA documentation [23]. Using traditional TCGA data that often have repeated whole genome sequencing (WGS) and RNA sequencing (RNA-seq) data for the same cases and sample types, Poore et al. produced microbial abundances after applying some quality filters to all available WGS and RNA-seq data. Since we focused mainly on microbial data, we extracted genomic samples present within the tumor microbiome samples for network construction. 8325 tumor microbiome samples and 6724 tumor genome samples from 18 tumor types were included in our analysis. 182 tumor microbiome modules and 570 mRNA modules were constructed. Correlation analysis between the microbial and mRNA modules was performed to identify OAM modules. Our study aimed to investigate the complex association between the tumor microbe module and the tumor genome module. To achieve this, we identified three tumor microbial modules and analyzed their relationship with the tumor genome. Firstly, we selected the module with the highest correlation with the tumor genome. The connections between tumor microbiome and tumor genome constitute many nodes on the heat map (Fig. 2B, Additional file 2: Fig. S2B, Additional file 3: Fig. S3). We then performed a cross-sectional count of nodes with statistical significance to identify the two microbial modules with the highest sum of correlation node counts. These three modules were considered as OAM (Fig. 1).

### Reactome enrichment analysis

The mRNA modules with the highest correlation to tumor microbiome modules in each tumor type were extracted to investigate the potential impact of tumor microbiome on the tumor genome. Enrichment analysis was performed using the “ClusterProfiler” R package and the “ReactomePA” R package for Reactome enrichment analysis [41, 42]. Gene enrichment analysis is an exploratory approach designed to generate hypotheses rather than test them, and overly strict p value thresholds may miss some biologically important and relevant pathways. In some cases, looser p value thresholds can improve the robustness and reliability of gene enrichment results [43–45]. Therefore, we think that choosing an adjusted p value of 0.2 is suitable based on the gene enrichment analysis results.

### ML methods

ML algorithms for tumor differentiation were trained, automatically tuned and tested using the “Caret” R package’s gradient boosting machine [46]. Gradient boosting machine is a ML technique that combines multiple weak learners, usually decision trees, into a strong learner by iteratively fitting the residuals of the previous learners and optimizing a loss function [47]. The microbiome within the OAM of each tumor was extracted to test the differentiation performance in 32 tumors. Separate, random selections of 70% and 30% of the data were used for training and testing, respectively. The data for each sample was centered and scaled to have mean zero and unit standard deviation during model training. Hyperparameter tuning optimization of the grid search was done by using twofold cross-validation. The gradient boosting machine and the subsequent ML hyperparameter tuning framework are described in Additional file 7. The final model performance, including the receiver operating characteristic (ROC) curves and confusion matrices, was generated by applying the final model to a 30% retention test set. The performance is evaluated using two metrics: Accuracy and the area under the curve of the receiver operating characteristic (AUROC). Accuracy is defined as the ratio of the number of correctly predicted samples to the total number of samples.

The mlr3 R package and its extension “mlr3proba” were used to build the ML algorithm for survival analysis [48, 49]. Genes within the highest correlation mRNA module and microiome in the OAM were included for patient prognosis prediction. Prognosis-related microbiome and mRNA were first extracted using univariate Cox regression, and microbiome and genes with p < 0.2 were considered statistically significant. The correlation-adjusted regression survival (CARS) score of prognosis-related microbiome were also calculated to characterize the prognostic value of microiome in OAM in pan-cancer, and the prognostic value of microbiome with the highest CARS score for pan-cancer was demonstrated by Kaplan–Meier (KM) curves [50]. The combined tumor microbiome and mRNA module was investigated for its prognostic ability by using four ML algorithms: “Coxnet”, “Random Forest”, “Xgboost” and “Coxboost” for the construction of prognostic models [51–54]. Coxnet fits a Cox proportional hazards model with elastic net regularization, which can deal with covariates that are correlated and have many dimensions, and estimate how they affect the hazard function. Random forest fits an ensemble of decision trees, which can deal with complex and non-linear relationships, and can reduce variance and improve accuracy. Random forest can also fit the random survival forests for survival analysis, an extension of the random forest algorithm that can deal with censored data. Xgboost fits tree-based models with gradient boosting, which can deal with various types of objectives, features, and distributions, and can be parallelized and distributed. Xgboost can also fit the accelerated failure time model for survival analysis, which assumes a linear relationship between the covariates and the log-transformed survival time. Coxboost fits the Cox proportional hazards model with gradient boosting, which can deal with covariates that change over time and censoring, and can perform variable selection and shrinkage, as well as estimate the baseline hazard function without a parametric form. All the above algorithms can handle high-dimensional and sparse data, and they are suitable for the microbiome and genome data. Their excellent predictive performance has been demonstrated in various previous studies [55–58]. Hyperparameter tuning of the grid search (Additional file 7: Table S3) was optimized by using fivefold cross-validation. Harrell’s consistency index (C-index) was used to evaluate the predictive power of the model, which was also generated by applying it to 30% of the test set. The predictive performance of the four ML algorithms in the test set was evaluated, and the ML algorithm with the highest C-index value was extracted as the best prognostic model for the tumor. The AUROC values at 1, 3 and 5 years were calculated using the “Time ROC” R package to evaluate the model.

The ability of the tumor microbiome to distinguish TP53 mutations was tested by incorporating 2511 microbial genera from 18 tumor types. A random forest ML algorithm was used to classify TP53 mutations based on the tumor microbiome. The dataset was split into 70% training and 30% test sets and scaled. The hyperparameters were optimized using a fivefold cross-validation grid search (Additional file 7: Table S3). The feature importance of the tumor microbiome was calculated to identify the most relevant microbe features for the model. The performance is evaluated using two metrics: AUROC and the area under the curve of the precision-recall (AUPRC).

### Immune infiltration analysis

Spearman correlation analysis was performed to extract Cluster of Differentiation (CD)8+ T cells and tumor-associated macrophages 1 (TAM1) cells in 18 types of tumors in Cibersort with OAM microbiome in the corresponding samples. A p-value of less than 0.05 was considered statistically significant.

### Statistical analyses

Statistical analyses were performed mainly using R (4.2.1), and wilcoxon rank sum test was used to compare group differences between groups. The survival distribution was estimated by KM survival curve analysis, grouped by the best cut-off value, and log-rank test was used for survival in different groups. The correlation between tumor immune infiltrating cell score and tumor microiome was evaluated by spearman correlation. A p value of less than 0.05 was considered statistically significant.

## Results

### WGCNA analysis suggests a correlation between tumor microbiome and tumor genome

We used the WGNCA analysis method to examine the correlation between tumor microbiome and the tumor genome. We selected a suitable soft threshold β, but the tumor microbiome network was less stable than the tumor genomic data in general (Fig. 2A, Additional file 2: Fig. S2A). The microbiome data of nearly half of the tumors we included in our analysis needed to be filtered based on raw counts to meet the needs of constructing a WGCNA network, especially for GBM (Additional file 5: Table S1).The correlation between tumor microbiome module and tumor genome module ranged from 0.12 (KIRC, p < 0.001) to 0.628 (KICH, p < 0.001) across all tumor types (Fig. 2B, Additional file 2: Fig. S2B, Additional file 3: Fig. S3). In 18 tumors, such as OV, HNSC, ESCA, STAD, GBM, etc., the tumor microbial module correlated with multiple modules of tumor mRNA. This indicates that the tumor microbiome may have a stronger association with the tumor genome in these tumors. Nevertheless, we have found some genomic modules in tumors other than BRCA, CESC, and OV that do not correlate with all microbial modules. Moreover, in renal tumors such as KIRC, KIRP, and KICH, this association was sparse but still significant. This suggests an interaction between the tumor genome and the tumor microbiome.

**Fig. 3:**
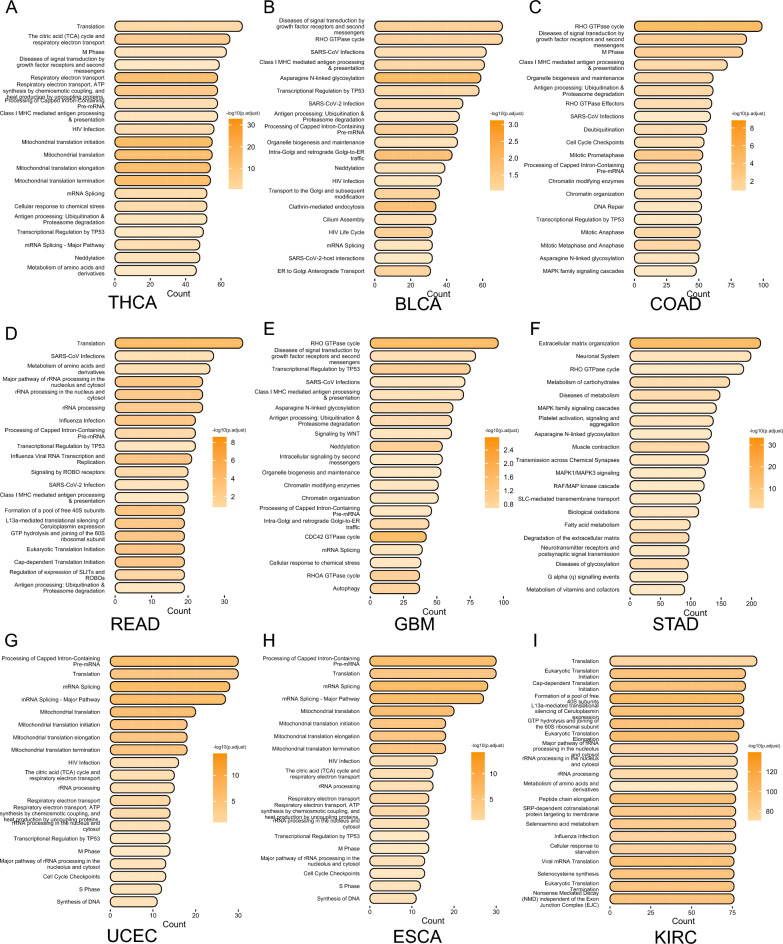
Reactome enrichment analysis results. **A**–**I** Reactome enrichment analysis results in THCA, BLCA, COAD, READ, GBM, STAD, UCEC, ESCA and KIRC. The x-axis shows the number of genes enriched in this term. The darker the color, the smaller the p value of the enriched term. Adjust p value < 0.2 was considered statistically significant. *THCA* thyroid cancer, *BLCA* bladder cancer, *COAD* colon cancer, *READ* rectal cancer, *GBM* glioblastoma, *STAD* stomach cancer, *CESC* cervical cancer, *ESCA* esophageal cancer, *KIRC* kidney clear cell carcinoma

To understand the main influences of the tumor microbiome on the tumor genome, we extracted the most relevant mRNA modules of each tumor for Reactome enrichment analysis (Fig. 3). Since WGCNA module identification was performed based on correlation in expression, we only found statistically significant pathway enrichment in a subset of tumors (adjust p < 0.2), but the pathways enriched to had many similar results. These mRNA modules were mainly enriched in infection, RHO GTPase cycle, Transcriptional Regulation by TP53,cell cycle, antigen presentation and mRNA modifications. Interestingly, we found enrichment of the Class I Major Histocompatibility Complex (MHC) mediated antigen processing and presentation pathway in THCA, READ, GBM, COAD, and BLCA. We also found that in STAD, it was mainly enriched in tumor stromal remodeling-related pathways. Based on the results of the pathway enrichment analysis, this suggests that OAM has a greater association with alterations in the tumor microenvironment.

**Fig. 4:**
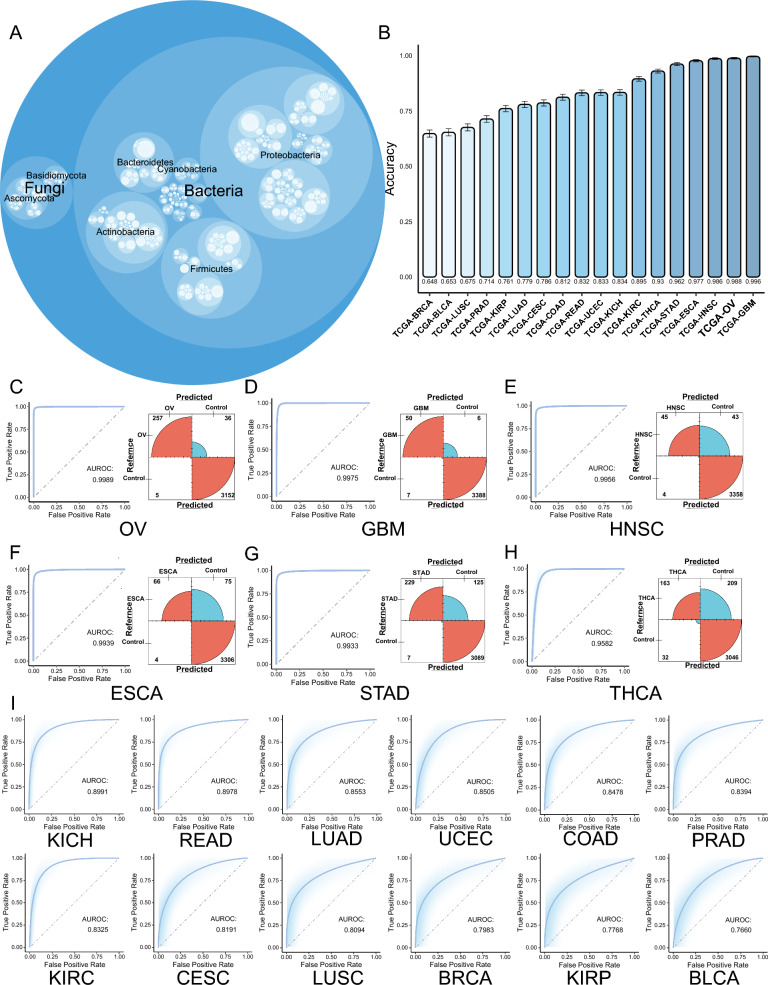
OAM is tumor type-specific. **A** Total composition of OAM in BRCA, BLCA, LUSC, PRAD, KIRP, LUAD, CESC, COAD, READ, UCEC, KICH, KIRC, THCA, STAD, ESCA, HNSC, OV and GBM. The size of the circle represents the number of OAM specie in that category. **B** Accuracy (y-axis) of ML models in BRCA, BLCA, LUSC, PRAD, KIRP, LUAD, CESC, COAD, READ, UCEC, KICH, KIRC, THCA, STAD, ESCA, HNSC, OV and GBM. **C**–**H** ROC curves and confusion matrix for the top six tumors with the highest AUROC. The red color in the confusion matrix represents correct predictions and the blue color represents incorrect predictions. The size of the arcs represents the number of correct predictions. **I** ROC curves for KICH, READ, LUAD, UCEC, COAD, PRAD, KIRC, CESC, LUSC, BRCA, KIRP and BLCA. *TCGA* the cancer genome atlas, *OAM* oncogenome associated microbiome module, *BRCA* breast cancer, *BLCA* bladder cancer, *LUSC* lung squamous cell carcinoma, *PRAD* prostate cancer, *KIRP* kidney papillary cell carcinoma, *LUAD* lung adenocarcinoma, *CESC* cervical cancer, *COAD* colon cancer, *READ* rectal cancer, *UCEC* endometrioid cancer, *KICH* kidney chromophobe, *KIRC* kidney clear cell carcinoma, *THCA* thyroid cancer, *STAD* stomach cancer, *ESCA* esophageal cancer, *HNSC* head and neck cancer, *OV* ovarian cancer, *GBM* glioblastoma, *ML* machine learning, *ROC* the receiver operating characteristic, *AUROC* the area under the curve of the receiver operating characteristic

To improve the accuracy of the analysis results due to the sparsity of the tumor microbiome data, we defined three correlated modules with the tumor genome in each tumor as OAM (Fig. 1). The OAM had the lowest number of microorganisms in THCA and READ (n = 264) and the highest number in ESCA originating from the Gastrointestinal (GI) tract (n = 1366, Additional file 6: Table S2). The OAM mainly consisted of bacteria, but also had a higher number of fungal species. The main phyla among bacteria were *Proteobacteria*, *Firmicutes*, *Actinobacteria*, and *Bacteroidetes*; and among fungi were *Ascomycota* and *Basdiomycota* (Fig. 4A).

### OAM is tumor type-specific

Previous studies have demonstrated that the intra-tumor bacteria and fungi are specific to each tumor type [3, 9, 33]. We used a gradient boosting machine ML approach to examine whether the OAM of different tumors is also tumor-specific at the pan-cancer level. The results showed that OAM could distinguish each cancer type from all others with varying degrees of accuracy (Fig. 4C–I). Overall, the area under the curve of the receiver operating characteristic (AUROC) was higher than 0.75 for all types of tumors, although the accuracy performed poorly in BRCA, BLCA, and LUSC (Fig. 4C–I).

We calculated the accuracy of the model and found that it had the highest accuracy in GBM (Accuracy = 0.996), followed by OV (Accuracy = 0.988), HNSC (Accuracy = 0.986), ESCA (Accuracy = 0.977), STAD (Accuracy = 0.962), and THCA (Accuracy = 0.930) (Fig. 4B). Similarly, in the calculation of the AUROC of the model, it also showed better differentiation of OV (AUROC = 0.999), GBM (AUC = 0.998), HNSC (AUC = 0.996), STAD (AUC = 0.993), ESCA (AUC = 0.994), and THCA (AUC = 0.958), indicating that OAM is highly specific in these six types of tumors with highly specific microbe-specific enrichment. We similarly computed the results for KIRC (Accuracy = 0.895, AUROC = 0.833), KICH (Accuracy = 0.834, AUROC = 0.899), UCEC (Accuracy = 0.833, AUROC = 0.851), READ (Accuracy = 0.832, AUROC = 0.898), COAD (Accuracy = 0.812, AUROC = 0.848), CESC (Accuracy = 0.786, AUROC = 0.819), LUAD (Accuracy = 0.779, AUROC = 0.855), KIRP (Accuracy = 0.761, AUROC = 0.777), PRAD (Accuracy = 0.714, AUROC = 0.839), LUSC (Accuracy = 0.675, AUROC = 0.809), BLCA (Accuracy = 0.653, AUROC = 0.766), and BRCA (Accuracy = 0.648, AUROC = 0.798) for accuracy and AUROC values (Fig. 4B, I). Combining these two metrics, in general, microbes in all types of tumors have some ability to discriminate between tumor types, suggesting that there is a tumor-type specific enrichment of OAM in some of these tumors, but that there may also be some microbes that are common between tumors.

Although it is difficult to avoid external contamination of TCGA data, the microorganisms of OAM still have some ability to distinguish between them by electronic decontamination and ML methods. This suggests that tumor microbiome may potentially interact with the host and lead to specific enrichment of tumor microbiome.

### OAM and the TME

Since the modules with the highest mRNA correlation were enriched to immune-related pathways in multiple tumors, we further analyzed the association between OAM and TME. Previous studies showed that intestinal bacteria can affect the ratio of TAM1/TAM2 cells in PAAD tumor-bearing mice [59], and that tumor microbiome can influence the infiltration of CD8+ T cells in the TME [7, 60, 61]. To further investigate which microorganisms in the OAM in various types of tumors affect the infiltration of CD8+ T cells and TAM cells in the TME, we therefore correlated the OAM with the CD8+ T cells and TAM1 cells in the corresponding samples. (Additional file 8: Table S4, Additional file 9: Table S5). Our study did not yield any statistically significant correlation between OAM and either CD8+ T or TAM1 in LUSC. Unlike the tumor genome, tumor microbiome had weak correlations with immune cells in the TME. The most correlated microorganism with CD8+ T cells was *Testudinibacter* in HNSC (r = 0.2912, p < 0.001, Additional file 4: Fig. S4A), and with TAM1 cells was *Granulicatella* in HNSC (r = 0.2797,p < 0.001, Additional file 4: Fig. S4B). These tumor microbiome may have immune-enhancing or suppressing effects. Although the OAM of each tumor was tumor type specific, we found that *Tissierella* had a strong positive correlation with both CD8 + T cells and TAM1 cells in PRAD and LUAD, and with TAM1 cells in BLCA. This suggests that *Tissierella* may be an immune-promoting microorganism that warrants further investigation.

However, we also found that *Megasphaera*, a microorganism with immunostimulatory effects in PAAD, had a negative association with CD8+ T cells and TAM1 cells in CESC (Additional file 8: Table S4, Additional file 9: Table S5) [62]. This indicates that the tumor microbiome may have different roles depending on the tumor type, similar to the genome. In the remaining types of tumors, the microbes with the highest correlation with CD8+ T cells are *Prevotellaceae* in KICH (r = 0.2158, p < 0.05), *Kushneria* in READ (r = 0.1978, p < 0.05), *Borrelia* in UCEC (r = 0.1926, p < 0.05), *Emcibacter* in ESCA (r = 0.1883, p < 0.05), and *Bifidobacterium* in BLCA (r = 0.1455, p < 0.05), which are positively correlated with CD8+ T cell infiltration scores. In contrast, *Paeniglutamicibacter* in BRCA (r = − 0.1051, p < 0.05), *Chryseolinea* in KIRC (r = − 0.1059, p < 0.05), *Corallococcus* in THCA (r = − 0.111, p < 0.05), *Gordonibacter* in COAD (r = − 0.1341, p < 0.05), *Oceanococcus* in OV (r = − 0.1632, p < 0.05), *Biostraticola* in STAD (r = − 0.1762, p < 0.05), *Blautia* in GBM (r = − 0.1884 1, p < 0.05), and *Virgibacillus* in KIRP (r = − 0.1903, p < 0.05) are negatively correlated. In the types of tumors with the highest correlation with TAM1 cells, *Tatumella* in KICH (r = 0.2671, p < 0.05), *Tetrasphaera* in ESCA (r = 0.2199, p < 0.05), and *Labedella* in UCEC (r = 0.1638, p < 0.05) are positively correlated with TAM1 infiltration scores. In contrast, *Orrella* in KIRC (r = − 0.0716, p < 0.05), *Mucilaginibacter* in BRCA (r = − 0.0841, p < 0.05), *Micromonospora* in THCA (r = − 0.1393, p < 0.05), *Gluconacetobacter* in STAD (r = − 0.1455, p < 0.05), *Riemerella* in CESC (r = − 0.1535, p < 0.05), *Lentzea* in COAD (r = − 0.1574, p < 0.05), *Chitinilyticum* in OV (r = − 0.1676, p < 0.05), *Senegalimassilia* in READ (r = − 0.1928, p < 0.05), *Pedobacter* in KIRP (r = − 0.2115, p < 0.05), and *Thioflexothrix* in GBM (r = − 0.2391, p < 0.05) are negatively correlated. These microbes that are correlated with TME are all worth further study.

### Combining OAM and tumor genome has value in predicting patient prognosis

**Fig. 5:**
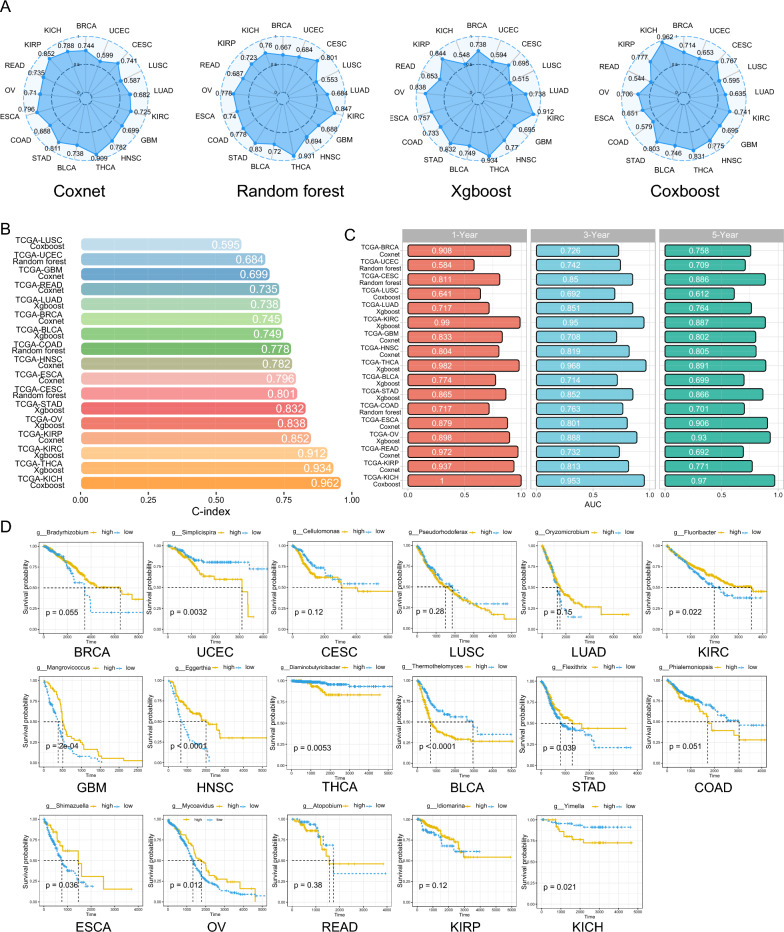
Combining OAM and tumor genome has value in predicting patient prognosis. **A** C-index of “coxnet”, “random forest”, “xgboost” and “coxboost” in BRCA, UCEC, CESC, LUSC, LUAD, KIRC, GBM, HNSC, THCA, BLCA, STAD, COAD, ESCA, OV, READ, KIRP and KICH. **B** C-index (x-axis) of the best model for in LUSC, UCEC, GBM, READ, LUAD, BRCA, BLCA, COAD, HNSC, ESCA, CESC, STAD, OV, KIRP, KIRC, THCA and KICH. Different tumor types are distinguished by different colors. **C** AUROC values (x-axis) of time-ROC at 1, 3 and 5 years for the best model of 17 tumor types. Red, blue and green represent 1 year, 3 years and 5 years respectively. **D** CARS score of the highest prognosis-related microorganisms in KM curves of 17 tumor types. The subgroups were distinguished by the best cut-off value and the log-rank test was used to calculate the p value. The high relative abundance group is shown in yellow and the low relative abundance group is shown in blue. *OAM* oncogenome associated microbiome module, *C-index* Harrell’s Consistency Index, *TCGA* the cancer genome atlas, *BRCA* breast cancer, *UCEC* endometrioid cancer, *CESC* cervical cancer, *LUSC* lung squamous cell carcinoma, *LUAD* lung adenocarcinoma, *KIRC* kidney clear cell carcinoma, *GBM* glioblastoma, *HNSC* head and neck cancer, *THCA* thyroid cancer, *BLCA* bladder cancer, *STAD* stomach cancer, *COAD* colon cancer, *ESCA* esophageal cancer, *OV* ovarian cancer, *READ* rectal cancer, *KIRP* kidney papillary cell carcinoma, *KICH* kidney chromophobe, *AUROC* the area under the curve of the receiver operating characteristic, *ROC* the receiver operating characteristic, *CARS* correlation-adjusted regression survival, *KM* Kaplan–Meier

Prognostic models that have been established so far tend to overlook the predictive value of the tumor microbiome in assessing the prognosis of cancer patients. It remains unclear how much prognostic value can be added by integrating the tumor microbiome with the tumor genome for patients with tumors. To address this gap, we integrated the OAM with highly correlated mRNA modules from the WGCNA analysis for subsequent microbiome-tumor genome prognostic modeling. We used univariate Cox regression to extract prognosis-related microbiome and mRNA data, with statistical significance assigned to those with p < 0.2 (Additional file 10: Table S6, Additional file 11: Table S7). We have obtained a large number of tumor microbes that have not been studied before and are associated with improved prognosis or increased risk in cancer patients.The KM curves indicate the prognostic value of the microorganism with the highest CARS score in each tumor (Additional file 12: Table S8, Fig. 5D). In our analysis, *Simplicispira* in UCEC, *Diaminobutyricibacter* in THCA, *Thermothelomyces* in BLCA, and Yimella in KICH were shown to be associated with poor prognosis in cancer patients (Fig. 5D). On the other hand, *Fluoribacter* in KIRC, *Mangrovicoccus* in GBM, *Eggerthia* in HNSC, *Shimazuella* in ESCA, and *Mycoavidus* in OV were shown to be associated with improved prognosis in cancer patients (Fig. 5D). We also found many microbes that are correlated with CD8+ T cells or TAM1, which can also be further validated in prognosis analysis for their research value. For example, *Williamsia*, a microbe that is negatively correlated with CD8+ T cells (r = − 0.0599, p < 0.05) and TAM1 (r = − 0.065, p < 0.05) in BRCA, was shown to be associated with poor prognosis in BRCA patients in univariate Cox regression with an hazard ratio (HR) > 1 (HR = 5.78 (1.46–22.86), p < 0.05, Additional file 8: Table S4, Additional file 9: Table S5, Additional file 10: Table S6). Similarly, another microbe, *Biostraticola*, which is negatively correlated with CD8+ T cells in STAD (r = − 0.1762, p < 0.001), was shown to be associated with poorer patient prognosis in univariate Cox regression (HR = 101.33 (4.49–2288.39), p < 0.05, Additional file 8: Table S4, Additional file 9: Table S5, Additional file 10: Table S6). Similarly, we also found microbes that are associated with improved prognosis in cancer patients. In OV, the protective microbe *Lottiidibacillus* (HR = 0.44 (0.24–0.79), p < 0.01) was shown to be positively correlated with CD8+ T cells (r = 0.0907, p < 0.05) or TAM1 (r = 0.0967, p < 0.01, Additional file 8: Table S4, Additional file 9: Table S5, Additional file 10: Table S6).

To fully assess the predictive power of the microbiome-tumor genome prognostic model, we used four ML algorithms—coxnet, random forest, xgboost and coxboost—to construct the model. We did not analyze the predictive value of the microbiome-tumor genome prognostic model in PRAD because the integration of tumor microbial data with mRNA data resulted in too few patients with PRAD deaths (n = 11). Overall, all four models showed better performance under the tuning framework we established, but with some heterogeneity (Fig. 5A). For instance, KICH’s features performed significantly differently in coxnet, xgboost and random forest compared to coxboost’s algorithm, even when using the same training and test sets. It has a C-index value of 0.962 in coxboost algorithm but 0.548 in xgboost algorithm (Fig. 5A). Therefore, we extracted the model with the highest C-index value for each tumor test set as the final model for that tumor (Fig. 5B). Unfortunately, despite using the best performing algorithm of the four, the microbiome-tumor genome prognostic model of LUSC performed poorly (C-index = 0.595). Under the coxnet algorithm based on our hyperparameter tuning framework, many tumors achieved good results. In GBM, READ, BRCA, HNSC, ESCA, and KIRP, the best C-index values in the test set were 0.699, 0.735, 0.745, 0.782, 0.796, and 0.852 respectively. Similarly, in the xgboost algorithm, the best C-index values for LUAD, BLCA, STAD, OV, KIRC, and THCA were 0.738, 0.749, 0.832, 0.838, 0.912, and 0.934 respectively. In the random forest algorithm, the best C-index values for UCEC, COAD, and CESC were 0.684, 0.778, and 0.801 respectively. The coxboost algorithm only achieved the best C-index values in LUSC and KICH tumors at 0.595 and 0.962 respectively (Fig. 5B). Additionally, we calculated the time-AUROC for each tumor (Fig. 5C), and found that overall, the best model had the best results for 1-year prediction. However, the AUROC value for predicting 1-year prognosis in UCEC is only 0.584, mainly showing the predictive value for long-term prognosis. Moreover, the AUROC for LUSC at 1, 3 and 5 years remained poor. The microbial-host prognostic models for the remaining tumors showed good prognostic values at 1, 3 and 5 years.

### Relationship between tumor microbiome and TP53 mutations

**Fig. 6:**
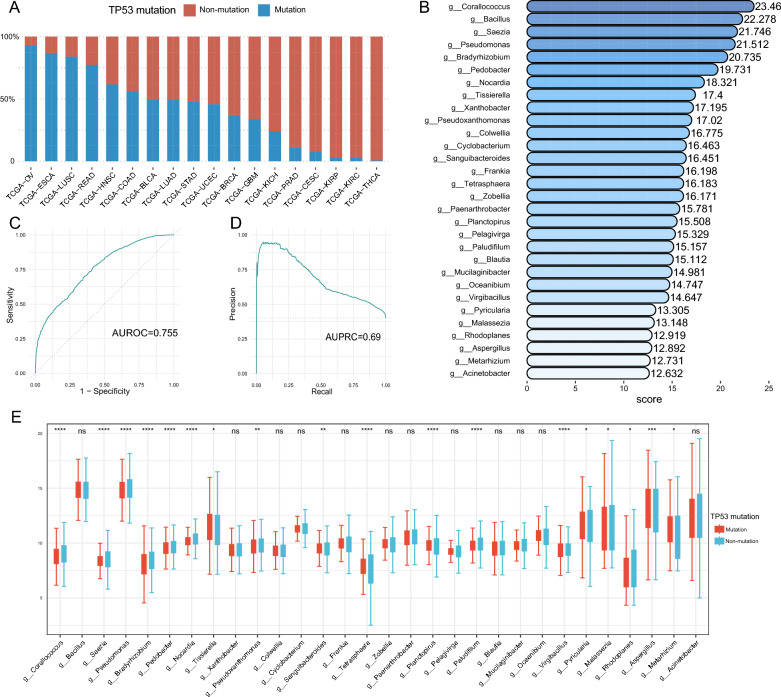
Tumor microbiome can distinguish TP53 mutations. **A** The proportion (y-axis) of tumors with TP53 mutations (blue) and TP53 non-mutations (red) in OV, ESCA, LUSC, READ, HNSC, COAD, BLCA, LUAD, STAD, UCEC, BRCA, GBM, KICH, PRAD, CESC, KIRP, KIRC and THCA. **B** Top 30 tumor microbiomes with highest ML model importance score (x-axis). The darker the color, the higher the importance score. **C** AUROC value of the ML model. **D** AUPRC value of the ML model. **E** Distribution of the top 30 tumor microbiome of highest ML model importance in TP53 mutations (red) and TP53 non-mutations (blue). The y-axis indicates the relative abundance of microorganisms. Statistical tests were performed using the rank sum test. *p < 0.05, **p < 0.01, ***p < 0.001, ****p < 0.0001,ns P > 0.05. *TCGA* the cancer genome atlas, *OV* ovarian cancer, *ESCA* esophageal cancer, *LUSC* lung squamous cell carcinoma, *READ* rectal cancer, *HNSC* head and neck cancer, *COAD* colon cancer, *BLCA* bladder cancer, *LUAD* lung adenocarcinoma, *STAD* stomach cancer, *UCEC* endometrioid cancer, *BRCA* breast cancer, *GBM* glioblastoma, *KICH* kidney chromophobe, *PRAD* prostate cancer, *CESC* cervical cancer, *KIRP* kidney papillary cell carcinoma, *KIRC* kidney clear cell carcinoma, *THCA* thyroid cancer, *TP53* Tumor Protein P53; *ML* machine learning, *AUROC* the area under the curve of the receiver operating characteristic, *AUPRC* the area under the curve of the precision-recall, *ns* no significance

At this stage, it remains unclear whether there is a causal relationship between tumor mutations and the specific enrichment of the tumor microbiome. But, the results of our gene enrichment analysis have suggested that the tumor microbiome may be able to influence the transcriptional regulation of TP53 in tumor cells (Fig. 3). Mutations in TP53, which plays a crucial role in regulating cell proliferation and differentiation, have significant implications for tumor development [63]. The frequency of TP53 mutations varied among the 18 tumors included in the study, with OV, ESCA, LUSC, and READ having mutation rates of over 75%, while KIRC, KIRP, and THCA had mutation rates of less than 5% (Fig. 6A). Studies have shown that the tumor microbiome in colorectal cancer can be distinguished from non-KRAS mutated samples by ML, suggesting a possible association between tumor microbiome and tumor mutations [64]. To investigate this association between the tumor microbiome and pan-cancer TP53 mutations, we examined 2511 tumor microbiome genus at the pan-cancer level. Our results showed that the TP53 differentiation model we constructed demonstrated some differentiation ability (AUROC = 0.755, AUPRC = 0.692, Fig. 6C, D).

We also assessed the importance of random forest model features and identified the top five tumor microorganisms in order of importance: *Corallococcus*, *Bacillus*, *Saezia*, *Pseudomonas*, and *Bradyrhizobium* (Fig. 6B). We found that among the microorganisms in the top 30 importance scores, a variety of microorganisms were likewise the highest correlated with CD8+ T cellss or TAM1 cells infiltration in the OAM of all types of tumors, such as *Tissierella*, *Corallococcus* and *Blautia* (Additional file 4: Fig. S4). We compared the distribution of the top 30 microorganisms in importance between TP53 wild type and mutant type, and found that not all species showed statistically significant differences (18/30) due to algorithmic variations. *Pyricularia* and *Aspergillus* are mainly enriched in TP53 mutations, while *Corallococcus*, *Saezia*, *Pseudomonas*, *Bradyrhizobium*, *Pedobacter*, *Nocardia*, *Tissierella*, *Pseudoxanthomonas*, *Sanguibacteroides*, *Tetrasphaera*, *Planctopirus*, *Paludifilum*, *Virgibacillus, Malassezia*, *Rhodoplanes* and *Metarhizium* are mainly enriched in TP53 wild type (Fig. 6E). Our findings demonstrate an association between the tumor microbiome and TP53 mutations, and microorganisms with high interpretation of the model may be potential targets for future tumor microbiology research.

### *Tissierella* may be a potential tumor suppressor-associated microorganism

**Fig. 7:**
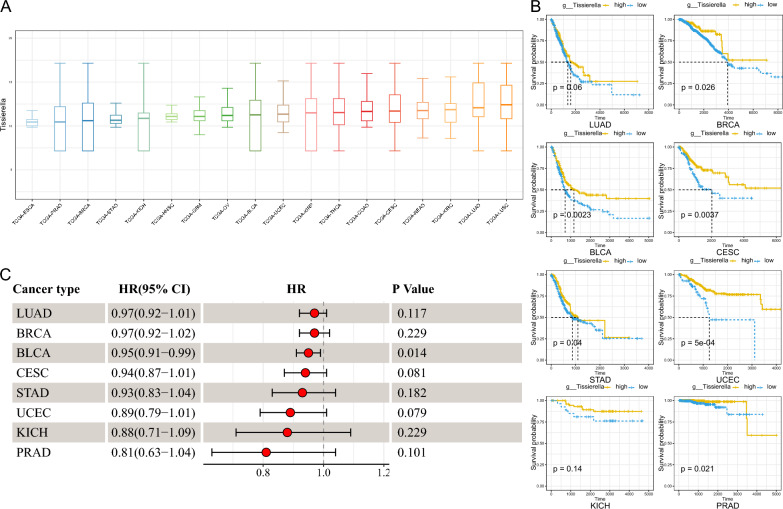
*Tissierella* may be a tumor suppressor. **A** Relative abundance (y-axis) of *Tissierella* in ESCA, PRAD, BRCA, STAD, KICH, HNSC, GBM, OV, BLCA, UCEC, KIRP, THCA, COAD, CESC, READ, KIRC, LUAD and LUSC. **B** Survival distribution of *Tissierella* high- and low-abundance groups in LUAD, BRCA, BLCA, CESC, STAD, UCEC, KICH and PRAD. The subgroups were distinguished by the best cut-off value and the log-rank test was used to calculate the p value. The high relative abundance group is shown in yellow and the low relative abundance group is shown in blue. **C** Univariate Cox regression results of Tissierella in LUAD, BRCA, BLCA, CESC, STAD, UCEC, KICH and PRAD. *TCGA* the cancer genome atlas, *ESCA* esophageal cancer, *PRAD* prostate cancer, *BRCA* breast cancer, *STAD* stomach cancer, *KICH* kidney chromophobe, *HNSC* head and neck cancer, *GBM* glioblastoma, *OV* ovarian cancer, *BLCA* bladder cancer, *UCEC* endometrioid cancer, *KIRP* kidney papillary cell carcinoma, *THCA* thyroid cancer, *COAD* colon cancer, *CESC* cervical cancer, *READ* rectal cancer, *KIRC* kidney clear cell carcinoma, *LUAD* lung adenocarcinoma, *LUSC* lung squamous cell carcinoma, *HR* hazard ratio, *CI* confidence interval

Given that *Tissierella* demonstrated a positive correlation with immune infiltration levels of CD8+ T cells and TAM1 cells (Additional file 4: Fig. S4), and was more abundant in TP53 wild type than TP53 mutant (Median 11.2 vs. 11.1, p = 0.019, Fig. 6E), we further examined its expression in pan-cancer and its prognostic significance. he relative abundance of *Tissierella* was highest in lung tumors, such as LUSC and LUAD, and lower in ESCA (Fig. 7A). Univariate Cox regression analysis revealed that *Tissierella* was a protective factor for tumor prognosis across various tumor types, particularly BLCA (HR = 0.95 (0.91, 0.99), p = 0.014, Fig. 7C). KM curves similarly showed a better prognosis in patients with a higher relative abundance of *Tissierella* in BRCA, UCEC, CESC, LUAD, BLCA, STAD, PRAD, and KICH (Fig. 7B). These findings suggest that *Tissierella* may be associated with improved patient prognosis and warrant further follow-up studies.

## Discussion

This study involved constructing 182 tumor microbiomes and 570 mRNA modules for each of 18 tumors using the WGCNA approach. Our goal was to investigate the potential association between tumor microbiome and tumor genome. In our study, we found a connection between tumor microbiome and the genome, which was not elucidated in previous studies. Due to the complexity of this connection, in terms of the tumor genome, we mainly explored the genes in the mRNA module with the highest correlation with the tumor microbiome module. Our gene enrichment results show that tumor microbiota may affect the host cell’s antigen presentation ability, TP53 transcription regulation, cell cycle, RHO GTPase cycle, infection and other pathways. The enrichment of infection-related pathways in various types of tumors is easy to understand. In addition, in STAD, the main enriched pathway is the TME matrix change-related pathway. This has been reflected in previous studies of PAAD. In PAAD, changes in the matrix structure seem to be accompanied by changes in the tumor microbiome structure, although this causal relationship is still unclear [65]. Moreover, previous studies have reported that the microbiome within breast cancer tumor cells can regulate the RhoAGTPase-Rock-actin cytoskeleton remodeling pathway, making cancer cells more resistant to mechanical stress and thus promoting tumor metastasis [11]. We have enriched the RHO GTPase cycle pathway in BLCA, COAD, GBM and STAD. This suggests that in addition to breast cancer, tumor microbiome of other types of tumors also have the potential to promote tumor metastasis.

We found enrichment of the Class I MHC mediated antigen processing and presentation pathway in THCA, READ, GBM, COAD, and BLCA. This suggests that tumor microbiome may affect the antigen presentation function of tumor cells. Recent research has proposed that the gut and intratumor microbiome can impact tumor progression by regulating host metabolism and immunity, leading to the definition of the immuno-oncology-microbiome axis [5, 66]. For instance, in a mouse model of PAAD, microbial ablation resulted in a decrease in immunosuppressive CD206+TAM2 in tumors, along with an increase in TAM1. Moreover, cell-free extracts from PAAD-carrying human host gut bacteria and *Bifidobacterium pseudolongum* present in human PAAD attenuated M1 polarization of macrophages and reduced the antigen-presenting capacity of TAM [59, 67]. Additionally, *F. nucleatum*, which is abundantly enriched in CRC, interacts with the immune cells inhibitory receptor expressed by tumor-infiltrating immune cells via the adhesion element Fap2 to suppress tumor-infiltrating T cell activity and protect tumor cells from immune cell attack [68–71]. Our research suggests that the connection between tumor microbiome and TME can be extended to a wider range of tumor types.

The results of pathway enrichment analysis and previous studies prompted us to further explore specific microbiota that are correlated with CD8+ T or TAM1 cells. Therefore, we conducted a correlation analysis of microbes in OAM with CD8+ T cells and TAM1 cells. Our research results provide a large number of microbiota in the OAM of various types of tumors that are correlated with CD8+ T cells and TAM1 cells. Many of these microbiota have not been reported in previous studies. Whatsmore, the results suggest that the role of tumor microbiome in the TME may not be limited to constructing an immunosuppressive microenvironment. Instead, tumor microbes may also play an immunostimulatory role, but further studies are needed to confirm this finding. For example, *Bifidobacterium* in BLCA is positively correlated with CD8+ T cell infiltration. Previous studies have shown that oral administration of *Bifidobacterium* in melanoma mice improves the efficacy of PD-L1 inhibitors and stimulates anti-tumor immunity [72]. The analysis of *Bifidobacterium* in tumors and CD8 + T cell infiltration provides a new direction for research on the anti-tumor mechanism of *Bifidobacterium*.

In the era of high-throughput omics, ML has become a well-performing method for research in this area [73]. Our study suggests the presence of tumor type-specific enrichment in OAM, providing new ideas for further investigating the relationship between the tumor microbiome and tumor genome, although the mechanism of tissue-specific enrichment remains unclear. We used OAM as well as tumor genomes to study their common prognostic value. Our research shows that the combination of tumor microbiome and host genome has good prognostic value, although it did not achieve ideal results in LUSC. Our research results indicate that the microbiome-genome prognostic model has good predictive value for short-term prognosis, but primarily shows predictive value for long-term prognosis in UCEC. Additionally, we identified a large number of microbiota in the OAM that are associated with the prognosis of tumor patients. In our analysis, *Simplicispira* in UCEC, *Diaminobutyricibacter* in THCA, *Thermothelomyces* in BLCA, and *Yimella* in KICH were found to be associated with poor prognosis in tumor patients, while *Fluoribacter* in KIRC, *Mangrovicoccus* in GBM, *Eggerthia* in HNSC, *Shimazuella* in ESCA, and *Mycoavidus* in OV were associated with improved prognosis. Furthermore, by combining the relationship between tumor microbiome and TME, it may be possible to further evaluate more meaningful microbiota. For instance, *Williamsia* in BRCA, *Biostraticola* in STAD, and *Lottiidibacillus* in OV.

While it is difficult to avoid the possibility of overfitting by using data from TCGA alone, our results suggest that the interaction between the tumor microbiome and the host may have a greater impact on tumor progression and prognosis of tumor patients. Identifying prognosis-related microbes could contribute to improving the prognosis of tumor patients. Similar to the different composition of the gut microbial community causing different phenotypes, the tumor microbiome can also have very different prognoses depending on their composition. For instance, the composition of the tumor microbial community of PAAD patients with long overall survival time is significantly different from that of patients with short overall survival time, possibly due to the increased infiltration of CD8+ T cells in TME [7]. However, in STAD, patients with high tumor microbial diversity seem to have a worse prognosis than those with low microbial diversity [74]. Our study likewise showed that different types of tumor microbes play diverse roles in possibly influencing patient prognosis. For example, *Mycoavidus* may act as a protective factor for OV, whereas *Thermothelomyces* is a prognostic risk factor in BLCA. Although the ability to directly utilize tumor microbiome for relevant studies is limited by the fact that most of them do not appear to be cultured from tumors in a simple manner, technologies such as high-throughput sequencing have made it possible to study the prognostic significance of tumor microbiome [75].

Somatic mutations in the TP53 gene are prevalent in human cancers, occurring in almost all types including OV, ESCA, CRC, HNSC, laryngeal, and lung cancers [76]. At present, the causal relationship between tumor mutations and the specific enrichment of tumor microbiota remains unclear. However, in colorectal cancer, ML algorithms can distinguish between KRAS mutated and non-KRAS mutated samples using tumor microbiome [64]. This indicates that there may be a connection between tumor mutations and tumor microbiome. As a gatekeeper of cell proliferation and differentiation, mutations in TP53 have a significant impact on the development and progression of tumors. Prior studies have shown that TP53 mutations, particularly in CRC, disrupt the epithelial barrier, allowing bacterial infiltration and disease progression [77]. In LUSC, certain microbial species were more abundant in TP53-mutated tumors, indicating a possible association between TP53 mutations and tumor microbial enrichment [78]. However, the relationship between pan-cancer TP53 mutations and tumor microbiota has not yet been elucidated by any studies. Our results showed some ability to distinguish TP53 mutations in the tumor microbiome at the pan-cancer level (AUROC = 0.755), but some components could not be clearly distinguished due to the quality of TCGA data and heterogeneity of tumor microbiome. We examined the microorganisms with the top importance of the random forest classifier and showed that there is indeed a difference in microbial composition between TP53 wild type and mutant tumors. The top five most important tumor microbiota are *Corallococcus*, *Bacillus*, *Saezia*, *Pseudomonas*, and *Bradyrhizobium*. We further examined the top 30 microbiota in terms of importance scores, and due to differences in algorithms, only 18 microbiota showed statistically significant differences. In addition, among these 18 microbiota with statistical significance, except for Pyricularia and Aspergillus, which are mainly enriched in TP53 mutations, the other microbiota are mainly enriched in TP53 wild type. However, this does not rule out the potential significance of microbiota that do not show statistical significance in distinguishing between TP53 mutations and non-mutations. For example, *Bacillus* has been shown to be associated with improved prognosis in PAAD [7].

*Tissierella* belongs to phylum *Firmicutes* and order *Clostridiales*, but its position in the lower ranks depends on the taxonomy used [30]. Studies on the relationship between *Tissierella* and tumors are lacking, but some studies have shown that *Tissierella* can produce Short chain fatty acids (SCFAs) [79]. SCFAs are beneficial metabolites of microorganisms, including acetic acid, propionic acid, and butyric acid [80–82]. SCFAs play an essential role in regulating host energy metabolism and the immune system. Supplementation with probiotics that produce SCFAs has been shown to inhibit the development of GI tumors [83–85]. Our study suggests the presence of an immunostimulatory effect of *Tissierella*, which may be through the production of SCFAs in a way that creates an immune-promoting TME, but experimental verification is needed. *Tissierella* is predominantly enriched in patients with non-mutated TP53, suggesting that TP53 mutations cause tumor development while altering the composition of the tumor microbiome to benefit their survival. The association of *Tissierella* with prognosis somewhat confirms this conjecture, but further verification is required.

There are limitations to our study that should be acknowledged. First, despite using in silicon decontamination, it was difficult to avoid contamination by factors such as the center of the sequencing plate in the TCGA data. Second, we only used TCGA data for constructing microbiome-tumor genome prognostic models and did not use external data to validate their efficacy, making it difficult to avoid overfitting. Partly because of the limitations of appropriate tumor type samples, sample size and completeness of clinical information of the samples (complete survival information), it is difficult to perform external validation at this stage. Third, regarding microorganisms with potential biological significance, such as *Tissierella*, we did not perform further experimental validation partly due to the challenges associated with obtaining and culturing microorganisms. Finally, as the TCGA dataset is limited to tumor patients and adjacent normal tissues, it poses a challenge to determine whether the relationship between tumor microbes and tumor genome also exists in the tissues of healthy individuals.

## Conclusions

To investigate the interaction between the tumor microbiome and the tumor genome, we employed the WGCNA approach to study the relationship between microbial and genetic modules. Our findings revealed that the tumor microbiome correlated with the tumor genome, and there was a tumor type-specific enrichment of OAM that correlated with CD8+ T cell and TAM1 cell infiltration in the TME. Combining tumor genome mRNA expression and relative abundance data of tumor microbiome allowed us to predict patient prognosis. Furthermore, our analysis demonstrated that tumor microbes could identify TP53 mutations. Lastly, *Tissierella* displayed potential suppressive effects on cancer in our study, but further research is necessary to confirm these findings.

### Supplementary Information

**Additional file 1: Figure S1.** PCA plots before and after correction of batch effect. A, B Sample distribution before correcting for batch effects. C, D Sample distribution after correction for batch effects. The x-axis represents the first principal component and the y-axis represents the second principal component. PCA: principal component analysis; RNA-seq: RNA sequencing; WGS: whole genome sequencing.

**Additional file 2: Figure S2.** Separate WGCNA networks were constructed for the tumor microbiome data and genomic data. (A) Network topology analysis of various soft threshold powers in KICH, KIRC, KIRP, LUAD, LUSC, PRAD, READ, UCEC and THCA in microbiome (left two columns) and genome (right two columns). The first column panel shows the scale-free fit index (y-axis) as a function of the soft-thresholding power (x-axis) in the microbiome data. The second column panel displays the mean connectivity (degree, y-axis) as a function of the soft-thresholding power (x-axis) in the microbiome data. The third column panel shows the scale-free fit index (y-axis) as a function of the soft-thresholding power (x-axis) in the genomic data. The forth column panel displays the mean connectivity (degree, y-axis) as a function of the soft-thresholding power (x-axis) in the genomic data. The selection of the soft threshold β is shown in Additional file 5: Table S1. (B) Correlation analysis between the microbial (y-axis) and mRNA (x-axis) modules in BLCA, BRCA, CESC, COAD and KICH. The circle size represents the correlation between the microbiome module and the genome module. A p value < 0.05 was considered statistically significant. Red, blue, and yellow circles represent p values less than 0.001, 0.01, and 0.05, respectively. WGCNA: weighted gene coexpression network Analysis; KICH: kidney chromophobe; KIRC: kidney clear cell carcinoma; KIRP: kidney papillary cell carcinoma; LUAD: lung adenocarcinoma; LUSC: lung squamous cell carcinoma; PRAD: prostate cancer; READ: rectal cancer; UCEC: endometrioid cancer; THCA: thyroid cancer; BLCA: bladder cancer; BRCA: breast cancer; CESC: cervical cancer; COAD: colon cancer.

**Additional file 3: Figure S3.** Correlation analysis between the microbial (y-axis) and mRNA (x-axis) modules in KIRC, KIRP, LUAD, LUSC, PRAD, READ, THCA and UCEC. The circle size represents the correlation between the microbiome module and the genome module. A p value < 0.05 was considered statistically significant. Red, blue, and yellow circles represent p values less than 0.001, 0.01, and 0.05, respectively. KIRC: kidney clear cell carcinoma; KIRP: kidney papillary cell carcinoma; LUAD: lung adenocarcinoma; LUSC: lung squamous cell carcinoma; PRAD: prostate cancer; READ: rectal cancer; UCEC: endometrioid cancer.

**Additional file 4: Figure S4.** Microorganisms with the highest association with CD8+ T cells and TAM1 cells in 17 tumor types. The length of the lollipop represents the size of the correlation between the microbe and the immune cell. Red represents positive correlation, while blue represents negative correlation. CD: cluster of differentiation; TAM1: tumor-associated macrophages 1.

**Additional file 5: Table S1.** WGCNA parameters selection. WGCNA: weighted gene coexpression network Analysis.

**Additional file 6: Table S2.** OAM and mRNA modules incorporated into the analysis. OAM: oncogenome associated microbiome module; mRNA: messenger ribonucleic acid.

**Additional file 7: Table S3.** ML hyperparameter tuning results. ML: machine learning.

**Additional file 8: Table S4.** The correlation of tumor microbiome with CD8+ T cells. CD: cluster of differentiation.

**Additional file 9: Table S5.** The correlation of tumor microbiome with TAM1 cells. TAM1: tumor-associated macrophages 1.

**Additional file 10: Table S6.** Univariate Cox analysis of the tumor microbiome.

**Additional file 11: Table S7.** Univariate Cox analysis of the tumor mRNA. mRNA: messenger ribonucleic acid.

**Additional file 12. Table S8.** CARS score for prognosis-related tumor microbiome. CARS: correlation-adjusted regression survival.

## Data Availability

The TCGA tumor microbiome datasets generated analysed during the current study are available in the github (https://github.com/knightlab-analyses/mycobiome). The TCGA tumor mRNA datasets generated analysed during the current study are available in the UCSC Xena database (https://xenabrowser.net/datapages/).

## Abbreviations

TME: Tumor microenvironment
BRCA: Breast cancer
PAAD: Pancreatic cancer
TP53: Tumor protein P53
*F. nucleatum*: *Fusobacterium nucleatum*
CRC: Colorectal cancer
DNA: Deoxyribonucleic acid
Kras: Kirsten rats arcomaviral oncogene homolog
WGCNA: Weighted gene coexpression network analysis
ML: Machine learning
TCGA: The Cancer Genome Atlas
mRNA: Messenger ribonucleic acid
OAM: Oncogenome Associated Microbiome Module
log-cpm: Log-counts per million
SNM: Supervised normalization
UCSC: University of Cingifornia Sisha Cruz
Cibersort: Cell-type identification by estimating relative subsets of RNA transcripts
ACC: Adrenocortical cancer
MESO: Mesothelioma
TGCT: Testicular cancer
UCEC: Endometrioid cancer
CESC: Cervical cancer
LUSC: Lung squamous cell carcinoma
LUAD: Lung adenocarcinoma
KIRC: Kidney clear cell carcinoma
GBM: Glioblastoma
HNSC: Head and neck cancer
THCA: Thyroid cancer
BLCA: Bladder cancer
STAD: Stomach cancer
COAD: Colon cancer
PRAD: Prostate cancer
ESCA: Esophageal cancer
OV: Ovarian cancer
READ: Rectal cancer
KIRP: Kidney papillary cell carcinoma
KICH: Kidney chromophobe
WGS: Whole genome sequencing
RNA-seq: RNA sequencing
ROC: The receiver operating characteristic
AUROC: The area under the curve of the receiver operating characteristic
CARS: Correlation-adjusted regression survival
KM: Kaplan–Meier
C-index: Harrell’s Consistency Index
AUPRC: The area under the curve of the precision-recall
TAM: Tumor-associated macrophages
CD: Cluster of differentiation
MHC: Major histocompatibility complex
GI: Gastrointestinal
HR: Hazard ratio
CI: Confidence interval
SCFAs: Short chain fatty acids
PCA: Principal component analysis
ns: No significance
