# Supplementary material for: Weighted gene coexpression network analysis and machine learning reveal oncogenome associated microbiome plays an important role in tumor immunity and prognosis in pan-cancer
Source: J Transl Med. 2023 Aug 12;21:537. doi: 10.1186/s12967-023-04411-0 (PMC10422781; doi:10.1186/s12967-023-04411-0)
Supplement: Supplementary file 1 — Additional file 1: Figure S1. PCA plots before and after correction of batch effect. A, B Sample distribution before correcting for batch effects. C, D Sample distribution after correction for batch effects. The x-axis represents the first principal component and the y-axis represents the second principal component. PCA: principal component analysis; RNA-seq: RNA sequencing; WGS: whole genome sequencing. [file 12967_2023_4411_MOESM1_ESM.pdf]

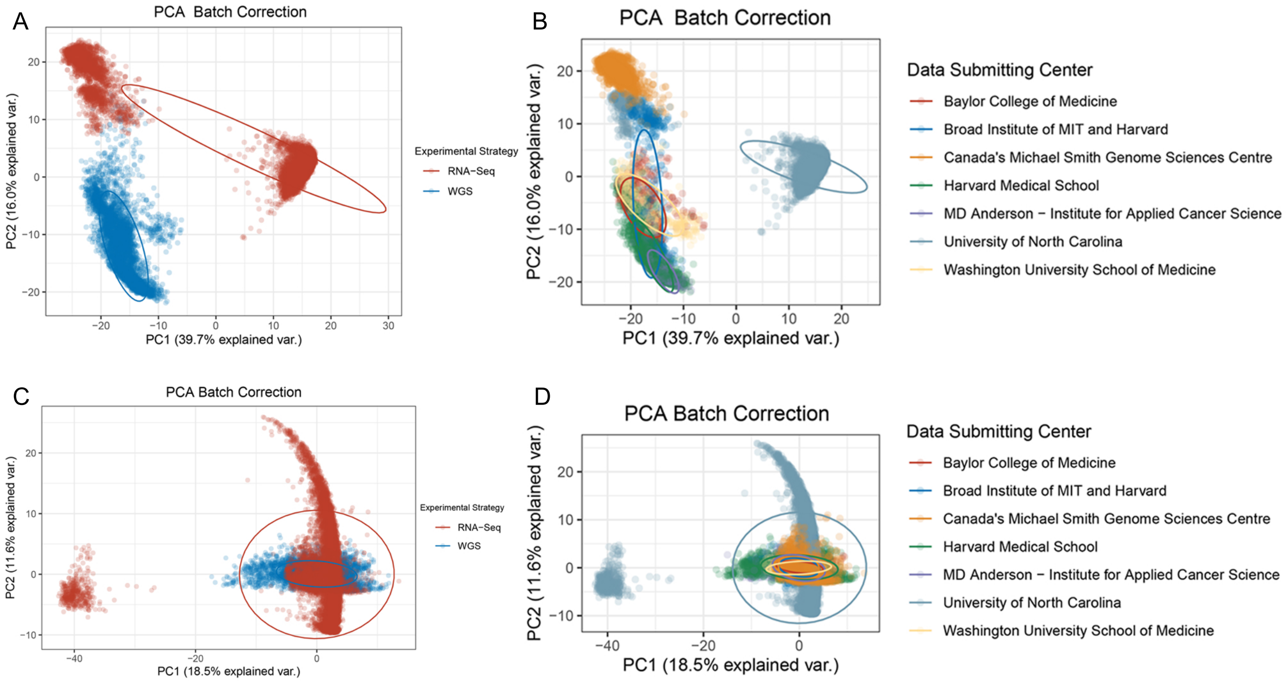

A
PCA Batch Correction
PC2 (16.0% explained var.)
PC1 (39.7% explained var.)
Experimental Strategy
RNA−Seq
WGS
B
PCA Batch Correction
PC2 (16.0% explained var.)
PC1 (39.7% explained var.)
Data Submitting Center
Baylor College of Medicine
Broad Institute of MIT and Harvard
Canada's Michael Smith Genome Sciences Centre
Harvard Medical School
MD Anderson − Institute for Applied Cancer Science
University of North Carolina
Washington University School of Medicine
C
PCA Batch Correction
PC2 (11.6% explained var.)
PC1 (18.5% explained var.)
Experimental Strategy
RNA−Seq
WGS
D
PCA Batch Correction
PC2 (11.6% explained var.)
PC1 (18.5% explained var.)
Data Submitting Center
Baylor College of Medicine
Broad Institute of MIT and Harvard
Canada's Michael Smith Genome Sciences Centre
Harvard Medical School
MD Anderson − Institute for Applied Cancer Science
University of North Carolina
Washington University School of Medicine
