# Supplementary figures and images for: Weighted gene coexpression network analysis and machine learning reveal oncogenome associated microbiome plays an important role in tumor immunity and prognosis in pan-cancer
Source: J Transl Med. 2023 Aug 12;21:537. doi: 10.1186/s12967-023-04411-0 (PMC10422781; doi:10.1186/s12967-023-04411-0)

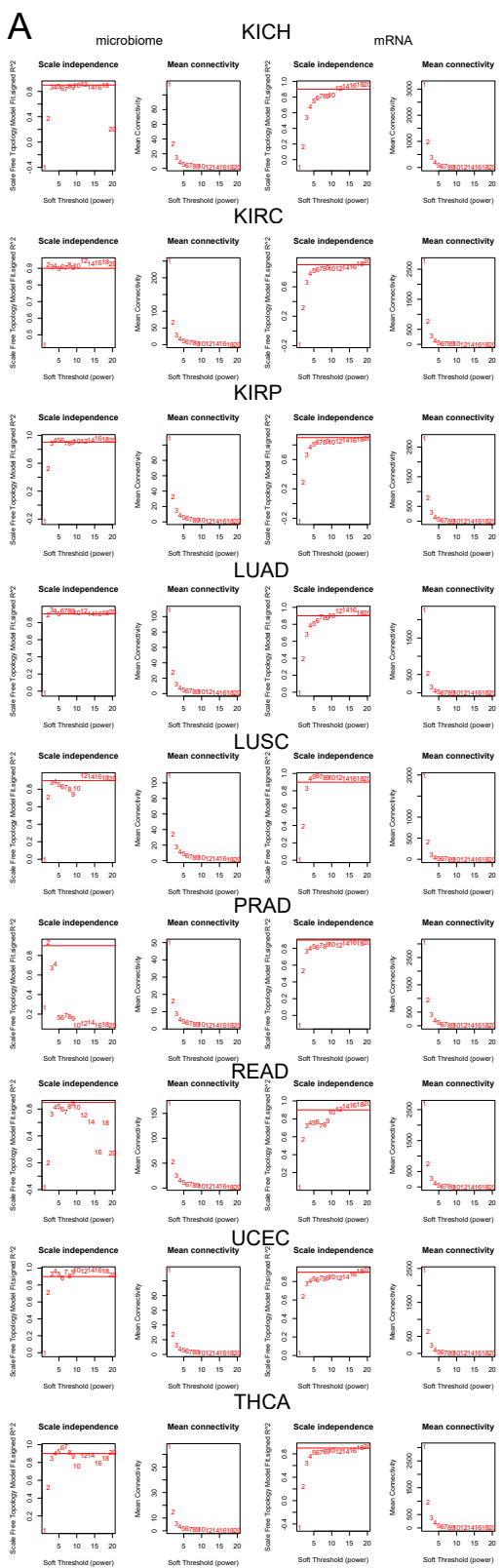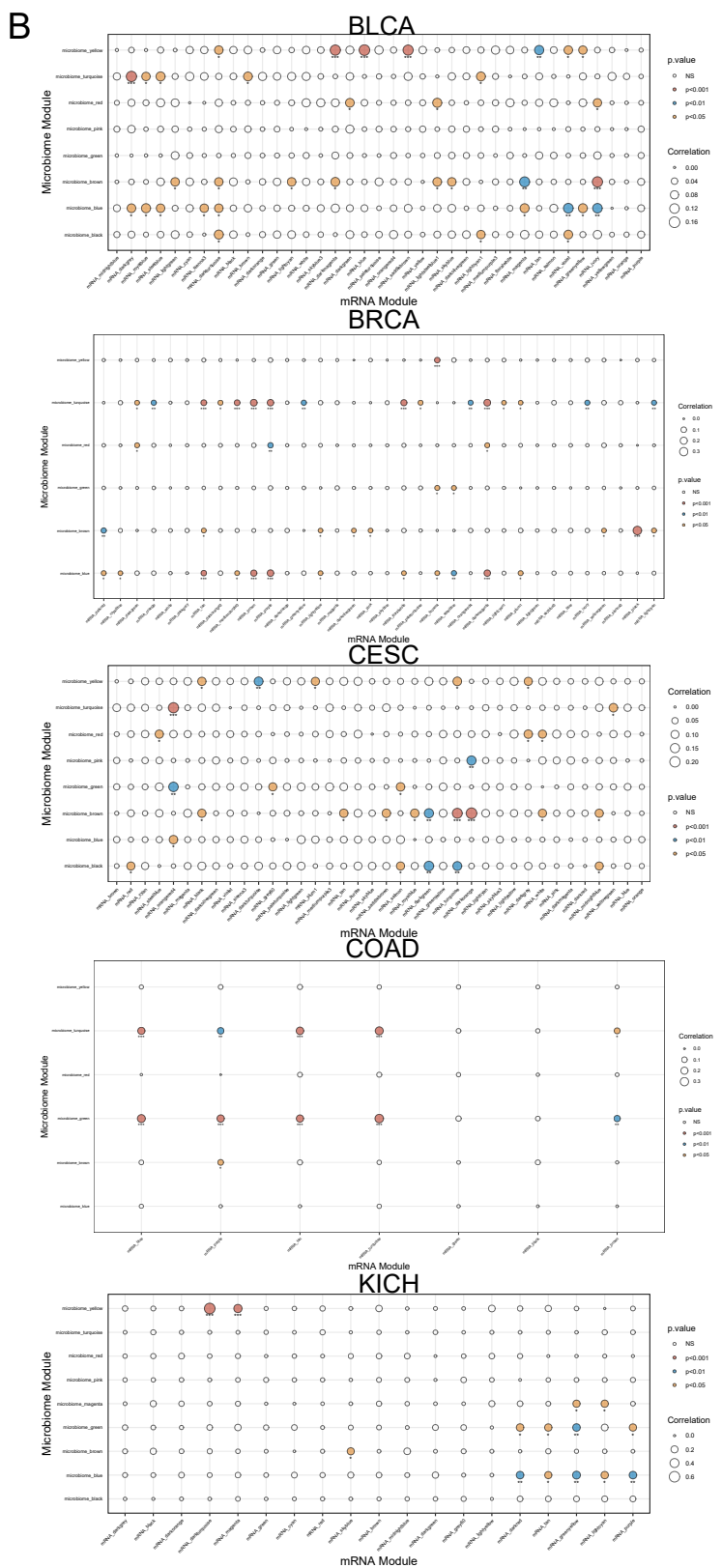

Supplement: Supplementary file 2 — Additional file 2: Figure S2. Separate WGCNA networks were constructed for the tumor microbiome data and genomic data. (A) Network topology analysis of various soft threshold powers in KICH, KIRC, KIRP, LUAD, LUSC, PRAD, READ, UCEC and THCA in microbiome (left two columns) and genome (right two columns). The first column panel shows the scale-free fit index (y-axis) as a function of the soft-thresholding power (x-axis) in the microbiome data. The second column panel displays the mean connectivity (degree, y-axis) as a function of the soft-thresholding power (x-axis) in the microbiome data. The third column panel shows the scale-free fit index (y-axis) as a function of the soft-thresholding power (x-axis) in the genomic data. The forth column panel displays the mean connectivity (degree, y-axis) as a function of the soft-thresholding power (x-axis) in the genomic data. The selection of the soft threshold β is shown in Additional file 5: Table S1. (B) Correlation analysis between the microbial (y-axis) and mRNA (x-axis) modules in BLCA, BRCA, CESC, COAD and KICH. The circle size represents the correlation between the microbiome module and the genome module. A p value < 0.05 was considered statistically significant. Red, blue, and yellow circles represent p values less than 0.001, 0.01, and 0.05, respectively. WGCNA: weighted gene coexpression network Analysis; KICH: kidney chromophobe; KIRC: kidney clear cell carcinoma; KIRP: kidney papillary cell carcinoma; LUAD: lung adenocarcinoma; LUSC: lung squamous cell carcinoma; PRAD: prostate cancer; READ: rectal cancer; UCEC: endometrioid cancer; THCA: thyroid cancer; BLCA: bladder cancer; BRCA: breast cancer; CESC: cervical cancer; COAD: colon cancer. [file 12967_2023_4411_MOESM2_ESM.pdf]

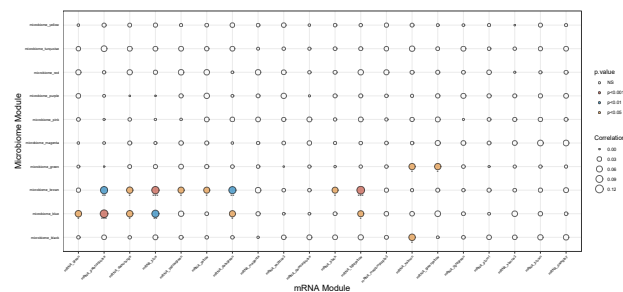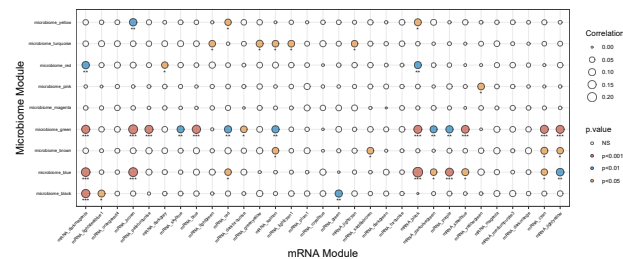

|  |  |  |
|--|--|--|
|  |  |  |
|--|--|--|

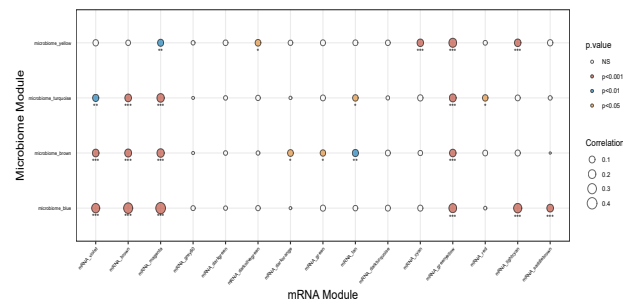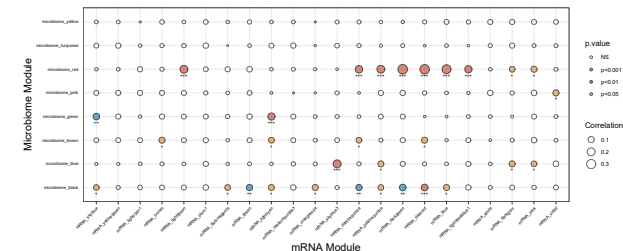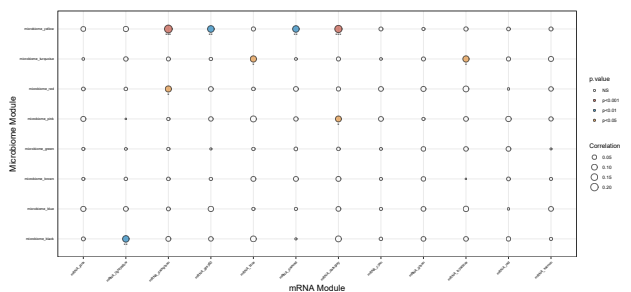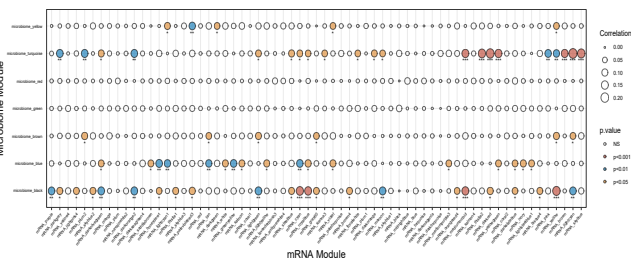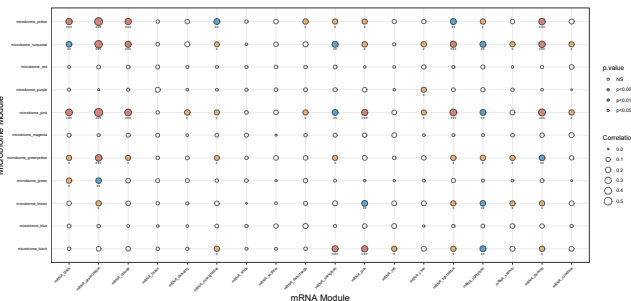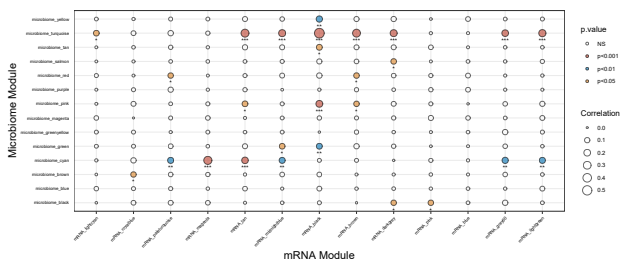

Supplement: Supplementary file 3 — Additional file 3: Figure S3. Correlation analysis between the microbial (y-axis) and mRNA (x-axis) modules in KIRC, KIRP, LUAD, LUSC, PRAD, READ, THCA and UCEC. The circle size represents the correlation between the microbiome module and the genome module. A p value < 0.05 was considered statistically significant. Red, blue, and yellow circles represent p values less than 0.001, 0.01, and 0.05, respectively. KIRC: kidney clear cell carcinoma; KIRP: kidney papillary cell carcinoma; LUAD: lung adenocarcinoma; LUSC: lung squamous cell carcinoma; PRAD: prostate cancer; READ: rectal cancer; UCEC: endometrioid cancer. [file 12967_2023_4411_MOESM3_ESM.pdf]

A

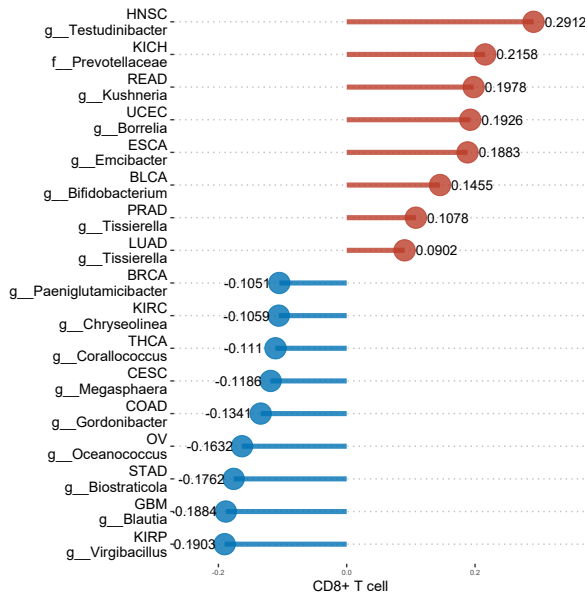

B

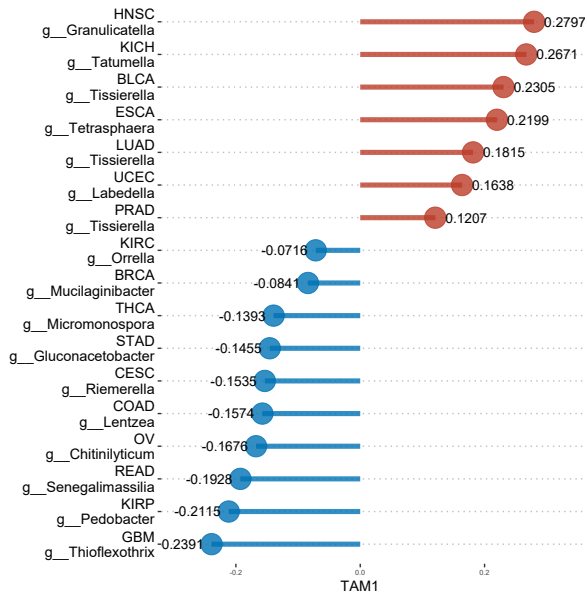

Supplement: Supplementary file 4 — Additional file 4: Figure S4. Microorganisms with the highest association with CD8+ T cells and TAM1 cells in 17 tumor types. The length of the lollipop represents the size of the correlation between the microbe and the immune cell. Red represents positive correlation, while blue represents negative correlation. CD: cluster of differentiation; TAM1: tumor-associated macrophages 1. [file 12967_2023_4411_MOESM4_ESM.pdf]
